# Editorial: Real World Outcomes of Lymphoma From India

**DOI:** 10.3389/fonc.2022.922370

**Published:** 2022-07-19

**Authors:** Lalit Kumar, Naresh KN, Sumeet Gujral, Padmaj Kulkarni, Martin R. Stockler, Reena Nair

**Affiliations:** ^1^ Department of Medical Oncology, All India Institute of Medical Science, New Delhi, India; ^2^ Department of Pathology, Fred Hutchinson Cancer Research Center, Seattle, WA, United States; ^3^ Department of Pathology, Tata Memorial Hospital, Mumbai, India; ^4^ Department of Medical Oncology, Deenanath Mangeshkar Hospital & Research Center, Pune, India; ^5^ Department of Medical Oncology, National Health and Medical Research Council (NHMRC) Clinical Trials Centre, University of Sydney, Sydney, NSW, Australia; ^6^ Department of Clinical Hematology, Tata Medical Center, Kolkata, India

**Keywords:** real world outcomes, diffuse large B cell lymphoma, biosimilars, targeted therapies, Hodgkin lymphoma, real world evidence, targeted therapy

Large number of cancers (>70%) are diagnosed in the low and middle income countries. The projected number of newly diagnosed lymphomas in India in 2020, was 11,230 and 41,607 for Hodgkin Lymphoma (HL) and Non Hodgkin lymphoma (NHL) respectively (1). The Age-standardized Incidence and Mortality ratio in Low/Medium High Development Index (HDI) countries is 4.0/2.5 for men and 2.8/1.7 for women per 100,000 population (2). Mortality is high in Low/Medium High Development Index (HDI) countries like India. The possible reasons include – limited access to tertiary cancer centers or lymphoma specialized centers, lack of trained lymphoma pathologists, and other socio-economic reasons for treatment discontinuation and early follow up drop outs.

Data on lymphoma outcomes is sparse and retrospective through chart reviews with inherent limitation (3). National Collaborative Groups established in Oncology, gather data from Institutes with Hospital Management System (HMS). Data maintained in HMS gives more reliable information of epidemiology, subtypes of lymphoma, outcomes of front line standard therapy and salvage therapy for relapsed and refractory lymphomas. A collection of articles on “Real World Outcome of Lymphoma from India” describes the clinical presentation, pathological subtypes and diagnostic capabilities, treatment practices for HL and DLBCL. These articles highlight the use of targeted therapies, rituximab and its biosimilars in DLBCL and Brentuximab Vedotin in relapsed HL, and its impact on the outcomes of curable lymphomas.

Onco-collect Lymphoma registry is a collaboration of 9 centers which maintain data of over 9000 lymphoma patients treated since 2011. The study published from this registry suggests DLBCL is the commonest subtype of lymphoma, with a median age of 57 years at presentation. Young age (< 65 years), early stage, low and low-intermediate International Prognostic Index (IPI), use of anthracycline and rituximab favorably impacted the 3-year event free survival (EFS) in a multivariate analysis. More patients receive standard chemo-immunotherapy (83.7%) with Rituximab, Cyclophosphamide, Hydroxyadriamycin, Vincristine and Prednisolone (R-CHOP) like therapies from 2011 onwards as compared to a decade back (37%) when most patients received CHOP- like therapy³. The cost-effectiveness of rituximab biosimilars and the availability of several biosimilars in India has reduced the price of rituximab and offers more value to the out-of-pocket spending by patients and charitable trusts (4). Real-world data from multiple Indian centers have confirmed the therapeutic triad of efficacy, tolerability, and safety of rituximab biosimilars (5–7).

Abbreviated chemotherapy cycles and omission of radiotherapy for early-stage DLBCL, negatively impacted the 3-year EFS. Present recommendation would be to limit the usage of abbreviated therapy to a highly selected group of DLBCLs with non-bulky disease, who undergo adequate staging with PET-CT scan at diagnosis and at response assessment. More real world evidence needs to be generated before the practice of reducing chemotherapy cycles or omitting radiotherapy in early-stage disease becomes a standard of care. This information assumes significance since access to salvage treatments is available for less than 50% of relapsed and refractory DLBCL. High dose chemotherapy with autologous hematopoietic stem cell transplant (AHSCT) was feasible in less than 10% adult patients in this series.

At diagnosis subtyping of DLBCL by Cell of Origin (COO) is not the standard of care. The capacity to perform immunohistochemistry (IHC) routinely to determine COO is limited to tertiary cancer hospitals in India. In an earlier study (71 patients), for DLBCL patients treated from 2015 to 2017, the 2-year disease free survival was 70% versus 53% (p=0.38) in Germinal Center B-cell (GCB) versus non GCB subtype. This study recommended the need to confirm the findings in a larger cohort of patients (8). GCB subtype determined by CD10 and bcl-6 positivity was available for less than half the patients from the OncoCollect registry. This study did not show an outcome difference for GCB and non-GCB. This may be the result of sub-optimal IHC classification of COO. The paper on Impact of cell-of-origin on outcome of patients with DLBCL treated with uniform R-CHOP protocol from North India from a large tertiary care institute, also reaffirms in a large cohort that the COO did not impact the outcomes of DLBCL.

Hemato-pathologists commonly face challenges while reporting on limited diagnostic tissues, especially on core biopsy specimens or overlapping morphological features, inadequate antibody panel etc. The use of multicolor flowcytometry (MFC) can simultaneously and objectively assess ≥8 markers, allowing detailed single-cell immunophenotyping even in limited tissue. It helps in a detailed evaluation of variation in the pan T-cell markers and variety of protein expression including clonality assessment of suspicious cells as detailed in the paper on Critical role of flowcytometric immunophenotyping of T-NK cell NHL from a tertiary cancer center.

The article on outcomes of adult HL from the OncoCollect multicenter registry suggests that the median age of presentation is 38 years, with half the patients presenting with B symptoms and more than half with advanced disease. Standard front line therapy adriamycin, bleomycin, vinblastine and dacarbazine (ABVD) was given to 95%. The 5-year EFS is 85.4% and 74.6% for early and advanced stage respectively and compares well with outcomes reported from single centers (9, 10). Of concern is significant drop in follow-up especially in the first two years post treatment. This increases the risk of advanced stage presentation at relapse. Second line therapy for relapsed and refractory HL was given to 75% of patients and 23% underwent the recommended high dose chemotherapy and AHSCT. Only a small proportion of patients received recommended targeted therapies with anti CD 30 monoclonal antibodies, and check point inhibitors in the relapsed setting. A single center experience with Brentuximab vedotin and bendamustine salvage suggests favorable toxicity profiles and long-term outcomes. This therapy was possible in a small group who could afford the treatment “out-of-pocket” and received Brentuximab on a named-patient drug access program. The outcomes compare well with the data from transitioned countries and represents a successful salvage option, albeit expensive, for an aspirational population of India.

Data maintained on a common software platform will help in finding innovative ways to complete standard treatment through patient access programs, and improve follow up in the first 2-year after completion of treatment. Increased availability of generic chemotherapeutic drugs, biosimilars and newer therapeutic modalities will further impact outcomes, and improve cure rates of lymphoma in India.

## Author Contributions

All authors listed have made a substantial, direct, and intellectual contribution to the work and approved it for publication.

## Conflict of Interest

The authors declare that the research was conducted in the absence of any commercial or financial relationships that could be construed as a potential conflict of interest.

## Publisher’s Note

All claims expressed in this article are solely those of the authors and do not necessarily represent those of their affiliated organizations, or those of the publisher, the editors and the reviewers. Any product that may be evaluated in this article, or claim that may be made by its manufacturer, is not guaranteed or endorsed by the publisher.
